# A Design Approach to IoT Endpoint Security for Production Machinery Monitoring

**DOI:** 10.3390/s19102355

**Published:** 2019-05-22

**Authors:** Stefano Tedeschi, Christos Emmanouilidis, Jörn Mehnen, Rajkumar Roy

**Affiliations:** 1 Manufacturing Department, Cranfield University, Cranfield MK43 0AL, UK; s.tedeschi@cranfield.ac.uk; 2 Design, Manufacturing & Engineering Management Department, University of Strathclyde, Glasgow G1 1XJ, UK; jorn.mehnen@strath.ac.uk; 3 School of Mathematics, Computer Science & Engineering, City, University of London, London EC1V 0HB, UK; r.roy@city.ac.uk

**Keywords:** industrial IoT, security, legacy production machinery, real-time condition monitoring

## Abstract

The Internet of Things (IoT) has significant potential in upgrading legacy production machinery with monitoring capabilities to unlock new capabilities and bring economic benefits. However, the introduction of IoT at the shop floor layer exposes it to additional security risks with potentially significant adverse operational impact. This article addresses such fundamental new risks at their root by introducing a novel endpoint security-by-design approach. The approach is implemented on a widely applicable production-machinery-monitoring application by introducing real-time adaptation features for IoT device security through subsystem isolation and a dedicated lightweight authentication protocol. This paper establishes a novel viewpoint for the understanding of IoT endpoint security risks and relevant mitigation strategies and opens a new space of risk-averse designs that enable IoT benefits, while shielding operational integrity in industrial environments.

## 1. Introduction

Industry 4.0 has a profound transformative effect on manufacturing environments, bringing in Internet of Things (IoT) connectivity to enable interaction that goes beyond basic machine-to-machine (M2M) communication. Such connectivity scales up the requirements of production data management and leads towards data-driven service innovation in manufacturing, wherein data analytics play a key role [1]. However, the potential arising from such enhanced connectivity is not sufficiently addressed in legacy production machinery, which is often poorly connected [2]. The connectivity capabilities of computer numerical controlled (CNC) machine tools remained constrained within the standardised CNC programming data exchanges, and further limited by a lack of versatile open application programming interfaces (APIs), making it difficult to monitor and control their functions within the whole production process [3]. CNC machine tools may already support a number of diagnostic services, which can be supplemented by additional sensors for direct or indirect monitoring. Such upgrades can be fitted within a networked factory environment through, making the machinery part of the Internet of Things (IoT) environment. IoT offers flexible means for connecting, as well as augmenting even modern machinery through advanced real-time data acquisition and monitoring services [4]. However, added connectivity brings in additional security and integrity risks for industrial environments. While security management has received extensive attention in the information security field, the functionality in production environments is delivered by the employed operational technology at the physical edge, and as such, its endpoint security deserves further attention. The potential operational impact that any security breaches may have on the integrity of industrial systems can be profound and need to be taken into account within the context of the targeted application domain at design stage.

**Figure 1:**
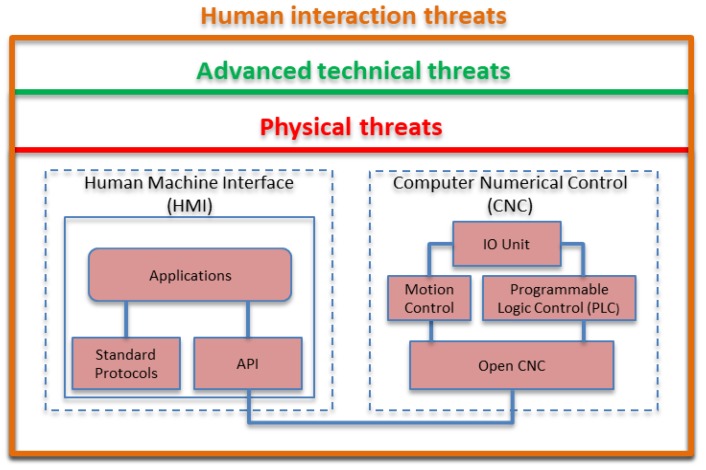
Abstract view of threats for legacy production machinery monitoring. API: application programming interfaces.

An abstract view of the nature of threats relevant to legacy production machinery is illustrated in Figure 1, showing physical threats at the lower layer, human interaction ones are at the top, and various types of technical threats in between. Physical threats involve actual physical tampering and may have direct tangible impact, for example causing physical damage on machinery, production, and infrastructure, or harm nearby personnel. Advanced technical threats refer to technology enabled access to different network layers and may involve data and software tampering. Human interaction threats are relevant to human interaction with technical systems.

While connected and smart environments are gradually implemented in healthcare, industrial, military, and transportation applications, security and privacy issues are increasingly highlighted as the major sources of risks. Industrial systems, in particular, strongly depend on preserving their physical and functional integrity, in additional to typical trust, identity, access control, and data protection through mechanisms, and require due consideration at the design stage of any networking upgrade to offer protection against multiple potential threats [5,6,7]. For example, permitting the cloning of tags or signal replaying [8,9] may allow attackers to gain access to critical data, services, and facilities. Information can be indirectly extracted from network, hardware, and software components, as some IoT systems may be susceptible to reverse engineering [10]. Defence techniques to prevent such attacks include cryptography [11], secure authentication protocols [12], improved resistance to cloning [13], and automatic malware detection [14]. However, such countermeasures are not included by design in typical industrial IoT endpoint devices, often either due to their resource-constraint nature, or through lack of appropriate designs, allowing such devices to be exposed to threats targeting real-time machine data access, tampering [15] with production machinery, modifying machining software or machine code, cloning devices, as well as initiating denial of service (DoS) or reverse engineering processes.

Various security approaches have been proposed to address or mitigate potential threats [16]. Established methodologies, such as STRIDE [17], first documented internally at Microsoft, involve threat identification and modelling as key activities, while others, such as PASTA [18] take a comprehensive application-oriented and risk-based perspective. While these methodologies have been successfully applied in practice, they do not offer higher-level guidelines and do not sufficiently address risks introduced at the endpoint-level of the IoT stack, considering the real-time application context and integration with industrial control systems (ICSs), such as supervisory control and data acquisition (SCADA), distributed control systems (DCS), and programmable logic controllers (PLC). Non-internetworked industrial control and monitoring systems were not vulnerable to cyber-attacks and there was limited or no clear direct physical integration between them and higher-tier enterprise systems [19]. In contrast, in modern cyber physical systems (CPS) and cyber physical production systems (CPPS) [20], supervisory control systems increasingly employ IoT connectivity to enable ubiquitous real-time monitoring [21], thereby also increasing the risk of cyber-attacks [22,23]. The mitigation of such risks needs to be introduced at the design or runtime stages [24,25]. Unsecured system operation may result in a real loss of service or loss of industrial environment control [26], often facilitated by obsolete versions of operating systems. The integration of IoT with ICS supports the aggregation of factory data to feed into SCADA and enterprise systems, giving rise also to new security challenges. The difference with previous generation SCADA systems is highlighted in Reference [27] where the need to handle endpoint security at the IoT device level is emphasised, which can be considered from a systems viewpoint, such as via SySML modelling of CPS agents [28]. The increasing incorporation of IoT in CPPS has motivated the development of assessment and identification techniques for CPS vulnerabilities, such as in the two-phased approach of Reference [29]. Specifically, phase one involves representing various processes via an intersection mapping of cyber, physical, cyber-physical, and human entities; and phase two introduces a decision tree-based structure for intuitive risk-based vulnerability analysis (e.g., low, medium, and high risk) [30], in a way that bears similarity to STRIDE and part of the PASTA methodology, but without the risk mitigation phase, demonstrating the approach on an automotive manufacturer case. IoT-enabled (or to this effect hybrid) SCADA systems employing wireless sensors network (WSN) may be vulnerable to external attacks. The impact of such threats to SCADA components needs to be analysed to prioritise risks [31].

In contrast with conventional information technology (IT), ICS are operational technology (OT) [32], acting to afford reliable real-time operations with required execution and safety properties at real production time. Security incidents have raised safety concerns in CPPS. Manufacturing enterprises have been the target of different cyber-attacks, aiming to acquire and gain access to sensitive information [33,34], or have fallen victims to ransomware operations targeting to block the computer access [35,36]. “Stuxnet” [37] was a notable worm attack which hit industrial PLC and SCADA vulnerabilities of nuclear plants, by being capable of periodically changing the frequencies of variable frequency drives, affecting centrifuge normal condition operation [38], even if the centrifuges themselves were equipped with cyber and physical security systems. In order to detect unexpected changes, enterprises often use quality control (QC) systems to alert for abnormal quality variations, However, these need to be both robust and strong in covering a range of relevant variations, and to be effective in this context they require threat analysis to better understand relationships between QC, manufacturing, and cyber-physical systems at design stage [39]. It is therefore, important to contextualise security approaches to the nature of CPS and ICS [40] and provide an analysis of security threat types and vulnerabilities, with an outline of security methods for attack prevention, detection, and recovery [41]. For example, in physical attacks, physical accessibility to the target device is by itself the prime vulnerability; data tampering with IoT networking, software attacks exploiting vulnerabilities inside IoT applications; and encryption attacks, involving breaking the system encryption are among the possible threats [42,43]. No security approach in CPPS environments would be sufficient without securing also the human interaction not only with computing and communication devices, but also with physical production assets, to mitigate functional integrity risks.

Focusing on IoT endpoints, device security can be supported by authentication mechanisms. For instance, identity-based authentication based on software defined networking (SDN) can target the distributed nature of wireless sensing in IoT, while consuming reduced resources, compared to public key cryptography (PKC) approaches [44]. Alternative authentication protocols diversify their approach between resource-rich and resource-constrained nodes. An example is the two-stage PAuthKey protocol, with a registration stage for obtaining cryptography credentials and an authentication step for establishing the communication [45]. Only resources-rich nodes communicate with the registration authority and the communication with constrained-resource nodes is then authenticated via implicit certificates. In practice, end-to-end security is applicable to the application layer, while lower layers rely on media access control (MAC)-tier security [46]. Overall, such an approach delegates security to edge nodes, enhancing resources efficiency. A hardware authentication method is presented in Reference [47], wherein each device is equipped with a unique fingerprint, consisting of multiple features, such as location, transmitter state, or physical object state. Alternatively, the authentication scheme is linked to distributed denial of service (DDoS) attack prevention via an algorithm that collects information from nodes to detect an attacker so as to prevent the working node from serving the malicious attacks [48]. In Reference [49], a dual authentication based on certificates and using datagram transport layer security (DTLS) between constrained IoT devices is proposed. An alternative approach uses a lightweight key agreement protocol to ensure anonymity, data secrecy, and trust between wireless sensor network (WSN) nodes in the IoT network [50]. In another network-centric approach, privacy invasion targeting networking patterns can be mitigated through synthetic packet-injection to hide real network traffic [51].

Considering that the intended functionality of CPPS is determined at the design stage, the same should be the case for IoT security in production environments, taking into account the potential sources of attacks and system vulnerabilities [52]. Therefore, understanding the nature and functionality of industrial systems is a prerequisite to designing their IoT security. With this in mind, after analysing and synthesising requirements for industrial systems, the Industrial Internet Consortium (IIC) has put forward the Industrial Systems Security Framework (IISF) [53]. The key differentiating factor between IISF and other IT or nonindustrial IoT security approaches lies in the joint handling of IT and OT. Security is viewed upon from the perspective of the potential impact on the delivered functionality of industrial systems, i.e., overall industrial systems’ trustworthiness. This is translated into a risk-based framework, directly linking security threats to risks arising from their impact on industrial systems trustworthiness. Recognising the ecosystem nature of IoT installations, IISF considers the whole system lifecycle and the permeation of trust across the system life-cycle phases and the system actors involved in them. IISF highlights the architecture view of IoT by considering security at the different layers of the IoT implementation stack, starting from the shop floor IoT end points. The shop floor end points include sensors, actuators, as well as connected production machines, which now become exposed to cyber-attacks, and therefore, lessons learned from IoT security need to be applied to develop strategies for networked production environments security. The IISF highlights the importance of the principle of isolation when securing IoT endpoints. This refers to process isolation within the operating system, container isolation implementing hardware or software-enforced boundaries, and virtual isolation protecting individual virtual instances of a trusted execution environment. However, endpoint security is still not sufficiently covered when upgrading legacy production equipment with IoT capabilities. This fundamental baseline of IoT endpoint security in industrial environment is, therefore, the target of the security thinking approach introduced in this paper, which includes:

- A novel risk-averse IoT endpoint security design thinking approach for industrial environments.
- An innovative IoT device security implementation of the design thinking approach, motivated by the isolation principle and applied at the interfaces between the key components of an IoT endpoint device and supported by a new lightweight authentication protocol with real-time features.
- Application of the above on a typical industrial case, that of production machinery monitoring.

The paper is organised as follows: Section 2 introduces the new endpoint security design thinking approach, comprising five stages. Section 3 analyses vulnerabilities when introducing IoT-enabled monitoring in manufacturing environments and introduces a relevant threats taxonomy, corresponding to the first two stages of the approach. Section 4 deals with stage 3 and employs an attack tree-modelling methodology to analyse security when introducing IoT in such environments, and presents steps taken to address them through adopting the subsystem isolation principle for an IoT data acquisition unit (DAQ). Section 5 implements stage 4 and applies the proposed approach on a legacy production machinery monitoring application. A representative implementation example case of the design solution, tested against a set of typical selected key attack types, is presented in Section 6, which corresponds to the final stage. Section 7 offers a discussion regarding limitations and further work, while Section 8 presents the conclusion.

## 2. Design Thinking for IoT Security in Industrial Environments

**Figure 2:**
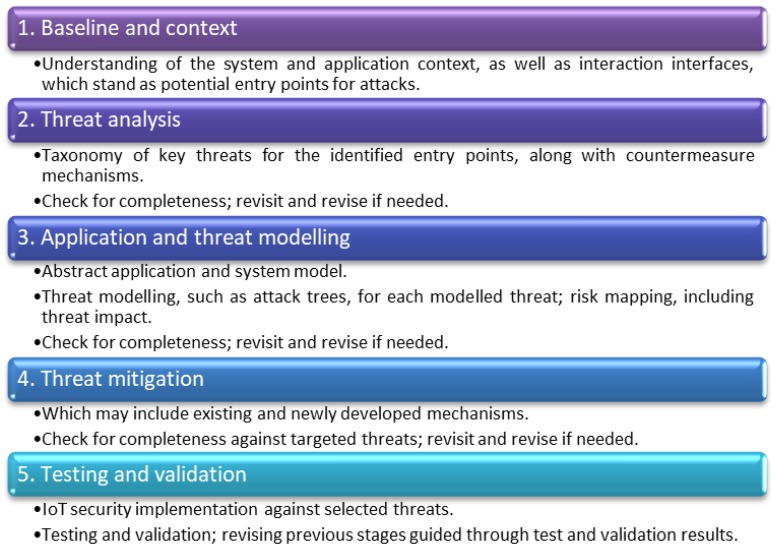
Design thinking approach for Internet of Things (IoT) device security.

Production machinery real-time monitoring is a major application target when introducing IoT in industrial environments and IoT endpoint devices are a fundamental component for any IoT security approach designed for such monitoring. Consistent with relevant recommendations and standards [54] and taking into account the nature of the manufacturing domain, the present research proposes a design thinking approach that clearly takes into account the application context and the context-specific potential impact of security compromises, a process more aligned with PASTA rather than STRIDE. The introduced systematic design thinking approach for IoT device security includes five key stages (Figure 2). Feedback from each phase may reveal a need to reconsider analysis, modelling, design, and implementation choices of all earlier phases.

(a) **Baseline and context**: This stage involves analysis of current practices in production environments. An understanding of the application context and system component interfaces, which may be exposed to security threats, is necessary to apply proposed concepts to a specific application target.
(b) **Threat analysis**: Having identified the high-level system interfaces which pose security risks, this stage involves an analysis of key security issues and vulnerabilities related to the implementation of IoT inside a production environment. Each vulnerability exploited by a threat can create an adverse impact on system integrity. A taxonomy of threats is produced, classifying them under the broader categories of physical, human interaction, and advanced technical ones. For each threat, possible mitigation mechanisms are proposed, and impact risk assessment is performed. Risk is quantified in three categories (High, Medium, and Low), consistent with recommendations [55].
(c) **Application and threat modelling**: The third phase provides the application context needed for an effective approach. It produces a more detailed model of the targeted system, along with its interfaces and functionality. Modelling tools include data flow diagrams (DFD) [56] to understand the permeation of data trust between components, and systematic threat modelling via attack trees [57], which need to be checked for coverage of security threats.
(d) **Threat mitigation**: The fourth phase deals with design and implementation of security threats-mitigation mechanisms. In the present work, an instance of the overall process is created and applied to the real-time monitoring application relevant to production environments.
(e) **Testing and validation**: This includes testing and validation of the mitigation mechanisms against selected threats. Testing may include simulation and functional testing, while validation may be performed in a test or a controlled operational environment. Results from functional and penetration testing can be fed back to improve the mitigation effectiveness. The functional aim of the test in the selected application case is to deliver uninterrupted real-time monitoring.

**Figure 3:**
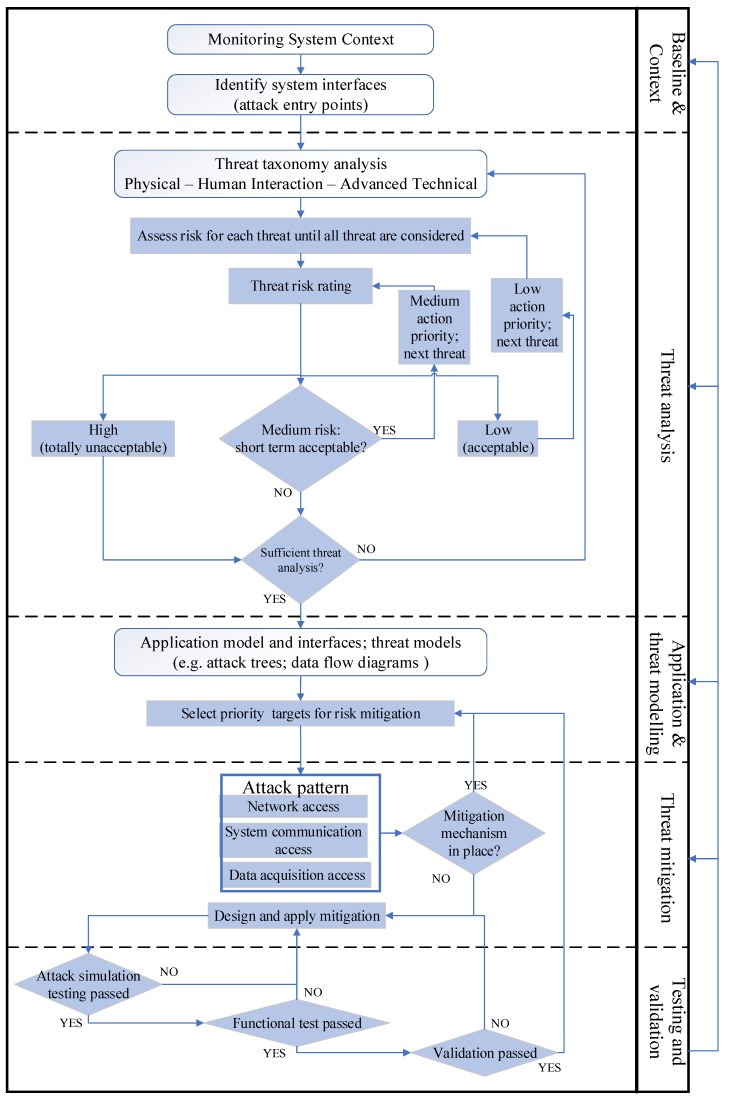
Design thinking for IoT device security in production environments.

Figure 3 shows a simplified flowchart for the proposed systematic approach applied to a real-time monitoring application relevant to production environments. For illustration purposes, this lists three types of attacks, namely network, system communication, and DAQ. These will be considered in more detail in the context of analysing the selected application case in Section 3 and Section 4, dealing with stages a, b, and c of the approach. To demonstrate the application of the new approach, the implementation and testing of mitigation mechanisms against denial of service (DoS) [58] and clone attacks [59] are presented in Section 5 and Section 6, corresponding with stages d and e. This involved the development of an innovative IoT endpoint device security implementation, introducing a new lightweight authentication protocol, consistent with the isolation principle and integrated in a prototype IoT DAQ device.

## 3. Monitoring Systems Security in Industrial Environments

### 3.1. Baseline and Context

The proposed design thinking approach is applied on a widely employed application in industrial environments, namely real-time condition monitoring (CM). CM refers to data acquisition and processing to infer the state of a machine over time [60]. It enables the identification of recommended maintenance actions based on the actual condition of monitored assets, rather than at predetermined intervals, thus allowing a condition-based maintenance (CBM) strategy to be implemented [61]. The determination of an appropriate CM approach consistent with a CBM strategy involves cost–benefits analysis, equipment audits, reliability and criticality audits, monitoring methods selection, data acquisition and analysis, determination of appropriate maintenance actions, and review processes [62]. A typical real-time condition monitoring system for legacy production machinery comprises sensors, a DAQ unit or microprocessor, computing resources, and adequate software [63], which may also be compactly available as a data-logging device. Signals acquired via the DAQ are processed by dedicated software, enabling the machine health to be determined. More advanced condition monitoring may also involve prognostics, and maintenance action determination. In wireless sensing, measurements can be acquired through a DAQ equipped with connectivity. Remote monitoring systems (RMS) [63] may already employ network communication between monitored machinery and back-end systems, or may involve retrofitting monitored assets with a communication device. RMS are applicable to both production processes and products. Connected products are amongst the prime developments which contributed to the concept of closed-loop product lifecycle management (PLM). IoT technologies not only enable product connectivity but also create data flows that upgrade the value proposition of product usage in operating environments [64,65]. Including IoT connectivity in such products creates additional vulnerabilities and this applies to IoT-enabled production machinery too. Therefore, the integration of IoT on legacy production machinery requires a rethinking of their security design [52].

**Figure 4:**
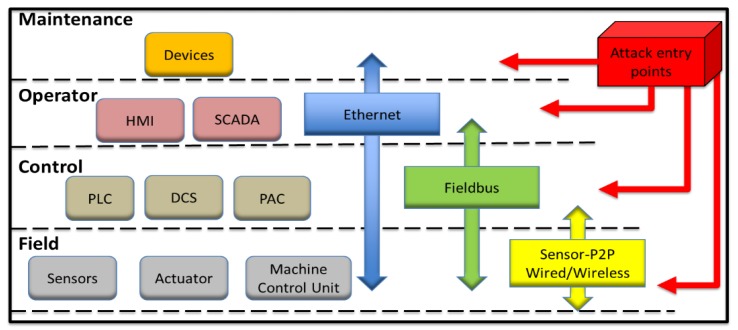
Legacy production machinery real-time monitoring system attack entry points. SCADA: Supervisory control and data acquisition; DCS: Distributed control systems; PAC: Programmable automation controllers; P2P: Peer-to-peer.

Figure 4 offers an abstract view of a machinery real-time monitoring system highlighting potential entry points for security attacks, assuming three standard communication types, namely wired or wireless device peer-to-peer (P2P), fieldbus, and Ethernet, as part of stage one of the approach. The networking enables data flows through sensors, PLCs, DCSs, programmable automation controllers (PAC), and human-machine interfaces (HMI), which in turn can drive recommendations for maintenance actions, and their planning and execution. This mapping can be looked upon from the viewpoint of the ISA-95 reference architecture, as adapted and mapped in five layers by the European Union Agency for Network and Information Security (ENISA) for the scope of smart manufacturing security [23]. Specifically, the field level of Figure 4 corresponds to Level 1, the control level to Level 2, the operator level to Level 3, and the upper-level refers to the application context, which in this case refers to interfaces exposed to devices accessing maintenance management and planning software and services, corresponding to Level 4. Unless the permeation of trust in such an architecture is duly considered, IoT-enabled industrial monitoring systems create increased security risks. Therefore, the additional focus is on the interfaces exposed to attacks, as per the first stage of the design approach of this paper. The next section provides an overview of threats analysis by threat type, applicable to industrial environments, relevant to stage 2 of the approach, while stage 3 in Section 4 studies in detail data interfaces and corresponding attack models for typical key security breaches in the studied problem, namely network, system communication, and DAQ access.

### 3.2. Threat Analysis

Threat analysis is the main activity in stage 2 of the process. The ISO27000 family of standards offers a broadly adopted framework for information security, including recommendations for information security management systems (ISMS, ISO/IEC 27001 [53]), where threat identification, as part of security risk assessment (ISO27005) [66], is central to devising a security approach. Vulnerabilities can be exploited by attack events to trigger security breaches, which, depending on the resulting sequence of events, may cause adverse impacts. Such impacts need to be translated into security risk mapping and quantification [67]. However, such recommendations are not specific enough to cover monitoring architectures for legacy production machinery. Approaches relevant to cloud security risk management [68] and lessons from other domains, such as finance, wherein cyber-attacks were already the prime sources of money loss, highlight the need to perform a domain-specific threat analysis to prevent adverse impacts [69]. Threat analysis is incomplete if it does not deal with application domain considerations. ENISA has produced a threat taxonomy for Industry 4.0 [23], which classifies threats into (a) nefarious activity or abuse; (b) eavesdropping, interception, or hacking; (c) physical attack; (d) unintentional or accidental; (e) failures or malfunctions; (f) outages; (g) legal; and (h) disaster. In this study, legacy production machinery and their monitoring systems define the application domain scope. Considering the focus on operational technology, physical types of threat are of prime concern [70]. Furthermore, considering the roles of personnel in production operations, a second key category would need to concentrate on human interactions. Finally, as advanced technology is involved in such manufacturing environments, compared to legacy production ones, technical threats is a natural third broad category. Therefore, the paper proposes that an appropriate high-level threat taxonomy should analyse human interaction (HIT), advanced technical (ATT), and physical threats (PT). Automatic operations are excluded from human–machine interactions and all operations that require human intervention (semiautomatic and manual) are included within human interactions. The software and network entry points are hard to enumerate and are subject to change. Entry points for hardware attacks are fewer and moderately well determined but attack targets can be diverse, targeting for example, information leakage [71], tampering [72], denial of service (DoS) [73], or cloning [59]. For each threat type, threat analysis needs to identify and describe activities which may allow the relevant vulnerability to be exploited (Table 1).

The potential harm that a threat may cause when exploiting vulnerabilities is assessed by rating the impact in categories such as those recommended in the National Institute of Standards and Technology (NIST) standard [55] (Table 2). Risk impact is linked to the functionality and integrity of the installation, and so risk analysis needs to consider its specific context. Risk levels can be adapted for a finer risk granularity if needed to serve specific application needs. The likelihood of the identified risks is then assessed (Table 3) and the final risk impact is quantified as the product of risk impact and likelihood (Table 4). IoT-enabled production assets create enhanced production data flows and therefore, DFD is a fitting model to study security vulnerabilities of key system entities. DFDs employ symbols for key processes and entities:

✓ External entities (EE), considered as end-point of a system;
✓ Processes (P), such as system or unit functionality;
✓ Data flows (DF), i.e., ways to transfer data;
✓ Data storage (DS), such as database or files for recorded information.

Finally, Table 5 offers a threat classification scheme along with risk impact quantification and applicable DFD modelling entities. Risks with high chance and impact are likely to occur, will have a significant impact, and should be given priority for mitigation. Risk quantification in the tables is indicative and actual risks in a specific implementation are likely to differ. An expert view of risk quantification in such industrial settings is available by ENISA [23]. This type of analysis is a necessary step to establish a sound baseline for designing a security approach to reduce risks for remote monitoring when upgrading legacy equipment with IoT devices. Having concluded with the stage 2 of the proposed approach, stage 3 aims to produce more detailed threat analysis for the targeted application domain, as described in the next section.

## 4. Application and Threat Modelling

### 4.1. Application Model and Data Interfaces

**Figure 5:**
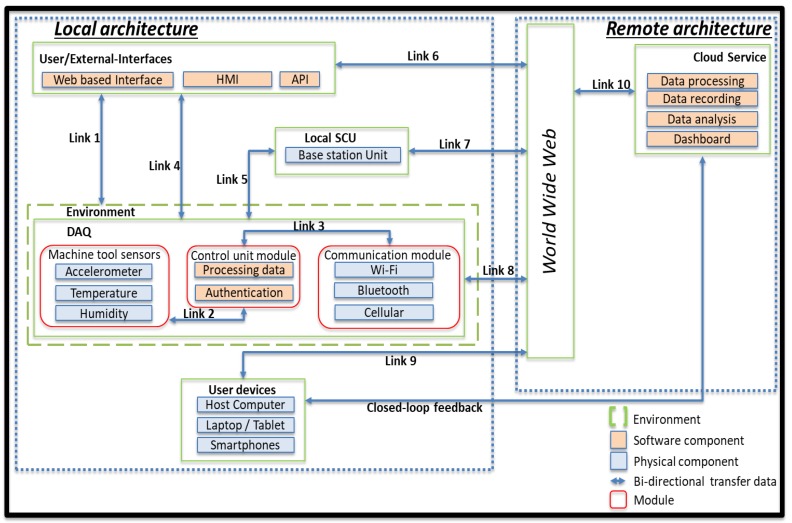
Abstract application model of the connected legacy production machinery. SCU: System control unit.

In the first part of stage 3 of the proposed approach, the application model considers key components of a machinery monitoring architecture and their data interfaces (links), to enable studying IoT security requirements in more detail. Following a representation similar to Reference [74], a simplified mapping of data exchanges is shown in Figure 5.

The local architecture includes the workplace with a legacy production machine and the IoT-enabled DAQ. The DAQ comprises three modules. The sensors module is physically attached to the machine. Collected real-time data are processed in the control unit module and can be passed to external or visual user interfaces. Transmitted data are sent to cloud-based systems, to the system control unit (SCU), or a mixture of both via the communication module. The remote architecture stores, manages, analyses, and visualises data on a dashboard to aid future actions. Such functionality is offered through the cloud to end-user devices, which can reside inside the local architecture. The data flows across the links are:

- Link 1: The environment includes the legacy production machinery, the DAQ modules, with access to configuration and management web services.
- Link 2: Data acquired from the sensor module are sent to the control module.
- Link 3: The control module manages the authentication process and passes data to the communication module.
- Link 4: The DAQ provides a user-interface to manage and visualise the data acquisition in real-time, residing within the monitored facility.
- Link 5: The DAQ and the SCU exchange data between the sensors and the local architecture.
- Link 6: Interfaces offer data visualisation and support or trigger appropriate actions.
- Link 7: The SCU employs cloud access to offer machine data management to users.
- Link 8: The DAQ communicates with cloud services via the internet.
- Link 9: User devices are communicating with the cloud or server through the internet, exchanging information relevant monitoring information.
- Link 10: Data management and visualisation services are made available to the user.

**Figure 6:**
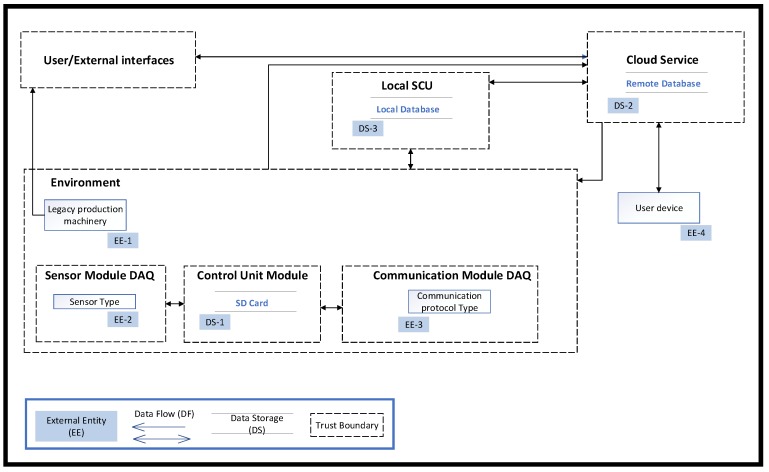
Data flow diagram of the connected legacy production machinery. DAQ: Data acquisition.

The mapped links are likely attack-entry points for the manufacturing environment. Link 1 is an entry point for physical threats, which can compromise the integrity of hardware devices, sensors, systems, and data. An attack can occur through connection to web-based interface, which is an entry point for software threats (e.g., viruses and trojans), as well as DoS and remote access control attack. Data interfaces require physical access to components, which can be exposed to cloning, side-channel, and reverse engineering attacks, but may also malfunction due to electromagnetic interference, voltage spike, and power fluctuation. Links 2 and 3 are entry points for physical intrusion and tampering, as well as cloning, side-channel, and reverse engineering. Links 4 and 5 are entry points for command injection, software attacks, DoS, and cloning, as well as unauthorised remote access. Links 6, 7, 8, and 9 are entry points for attacks causing a network breakdown or system process malfunctions through ATTs, such as DoS, command injections, reverse and social engineering attacks. Link 10 requires access credentials and is an entry point for error and omission, unauthorised remote access, social engineering, command injection, DoS, and software attacks. HITs are relevant to all interfaces and components and may cause data loss, process malfunctions, and network breakdown. Having available an abstract application and data exchange model helps towards application-specific threat modelling. DFDs between subsystems create an understanding of the permeation of trust between boundaries. Figure 6 shows a simplified DFD for IoT-enabled monitoring. Dotted line rectangles denote trust boundaries of subsystems; solid line rectangles represent external subsystems; arrows indicate data flows; interfaces with external entities and storage are marked with a solid coloured rectangle (not part of DFD). Data flows and trust boundaries constitute intermediate attach goals, and their modelling is the subject of the next section.

### 4.2. Threat Goals Modelling

The second part of stage 3 of the proposed approach deals with detailed threat modelling. In order to devise mitigation mechanisms, it is of interest to further understand specific goals that an attacker may set in pursuing attack targets. Focusing on the two high-risk priority attack threats (Table 5), namely DoS and cloning, it is of interest to study potential attack intentions and consequences. The main goals are: Gaining network or access, communication access to the supervisory and control architecture [75], and modifying the DAQ [76]. The potential impacts of these goals are analysed in Table 6, consistent with the reliability-oriented approach FMEA (failure mode and effects analysis). Specifically, impacts could affect different functions, which in the case of a production machine could be stated as [62]: P: Primary, affecting functions required to fulfil the machinery intended output (e.g., production of an item); S: Secondary, supporting the primary function (e.g., managing coolant in a machine tool); C: Control and protective, affecting the ability to control a process (e.g., adjusting feed rate in machining) or protecting workers, equipment, or the environment (e.g., stopping machining after tool breakage); I: Information, affecting ability to provide monitoring information for a function (e.g., failure to provide or display temperature reading); and U: Interface, affecting the interaction interface between two items. This makes the understanding of the potential consequences of an attack more tangible and aids the design and development of impact mitigation. The attack goals are next modelled, for example, via attack tree modelling, which is a common structured approach to illustrate in a logical way the main goals of an attacker. The top tree node is a key attack target. Lower level goals and individual malicious activities, which may contribute to reaching that goal, are located below the main node. Steps between the lower nodes and the top node depict intermediate states or attacker subgoals. This modelling is now applied for the machinery monitoring application, defining attack trees for the identified threats (e.g., Table 1 and Table 5) and specifically for each of the attack goals of Table 6.

#### 4.2.1. Network Access

**Figure 7:**
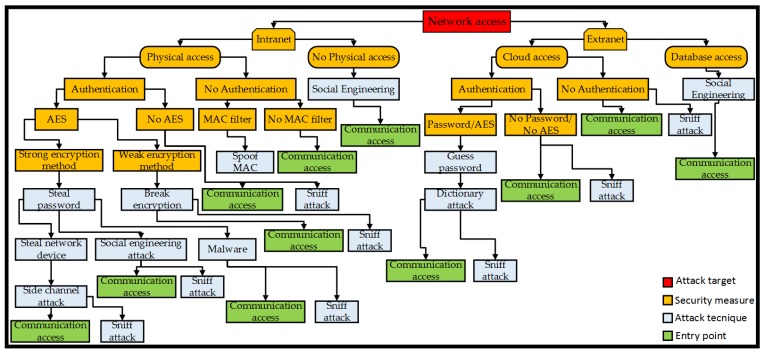
Network access attack tree. MAC: Media access control; AES: Advanced Encryption Standard.

Gaining access to the network wherein the monitoring system operates, an attacker can use malicious or fraudulent actions to gain access to data devices or server systems connected with the network. Figure 7 shows the attack tree that models the network access threat goals. Typically, enterprises may have a private and public network. Subject to access rights, these are exposed to personnel, customers, partners, or suppliers. Within the intranet there may be parts of the architecture which can be modified by access to the hardware for upgrading firmware, updating software, and replacing components, whereas other entities do not require physical access to the architecture and are only modified remotely. Upon gaining physical access to the hardware, the attacker can further access the network through devices, cables or ports, radio interference, or wireless and wired networking means. Without physical access, network access can be achieved via social engineering [77]. While encryption and a media access control (MAC) filter can be applied as security measures, spoofing attacks [78] can still be used to gain access to the network. An attacker can bypass strong encryption methods (such as Pretty Good Privacy (PGP) or Advanced Encryption Standard (AES)), by obtaining the encryption password mostly through social engineering, installing some malware for reading the password, or by breaking into specific network devices via a side-channel method. If the system devices are equipped with a weak encryption method, it may be easily broken with cryptography attacks. On the extranet side, the system can be equipped with password authentication. An attacker can use the dictionary method to guess the password, then bypass the firewall and gain access to the local network.

#### 4.2.2. System Communication Access

**Figure 8:**
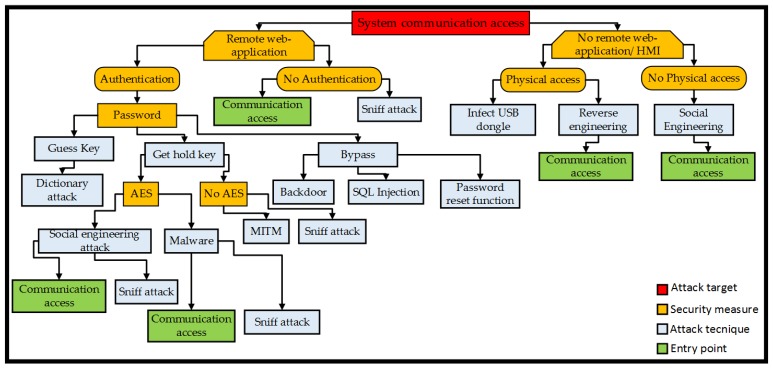
System communication access attack tree. MITM: Man-in-the-middle.

Remote access applications allow ubiquitous supervision and control through networked devices, whilst HMIs allow enable control via a front machine panel. An attack may seek to gain access to the communication system to compromise supervisory systems and modify machine or process parameters. An attack tree analysis for this threat goal is shown in Figure 8. If there is no authentication requirement, an attack can easily succeed in gaining access. When authentication is enforced, an attack may guess the access key by the dictionary method [79], or bypass the password using a backdoor secret method, such as chipset, cryptosystem, and an algorithmic structured query language (SQL) code injection [80]. When encryption is employed, the attack can obtain the key through a social engineering method or malware injection. Systems without encryption are susceptible to man-in-the-middle (MITM) method, where the attacker can spoof the system identity, waiting for a user to login and then save the credentials for future access. If physical access to the HMI is gained, the attacker can use an infected USB dongle to compromise the control system or employ reverse engineering to gain communication or achieve this without physical access via social engineering.

#### 4.2.3. Data Acquisition (DAQ) Access

**Figure 9:**
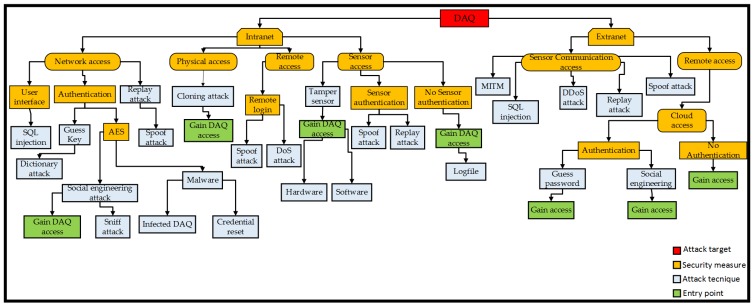
DAQ access attack tree. SQL: Structured query language.

Figure 9 displays the attack tree to acquire access to the DAQ. The side-channel method is one of the simplest physical access methods, allowing DAQ access to make modifications, such as install new firmware or patch, or replace hardware components. Using the network, the attacker can use SQL injection [81] to gain access to user devices or gain authentication to infect the DAQ with malware, and through the replay attack to spoof data. An attack can target DAQ access after remotely logging in with credentials to launch a DoS attack and flood available bandwidth. Accessing the sensors, the attacker can compromise hardware or software components to affect normal DAQ operation. When sensor authentication is not employed, an attack can gain DAQ access using log files to spoof data. From the extranet, an attack can gain DAQ access via the MITM method, SQL injection, or spoofing sensor information, replay attack method, or flood its connection via DoS. Alternatively, an attacker can remotely gain authentication to the cloud service and control the DAQ from there.

Attack tree modelling is a structured methodology for analysing security to drive the design and implementation of appropriate mitigation mechanisms. The next section takes into account such analysis to develop and test IoT endpoint device security for legacy production machinery monitoring.

## 5. Threat Mitigation for IoT-Enabled Production Machinery

**Figure 10:**
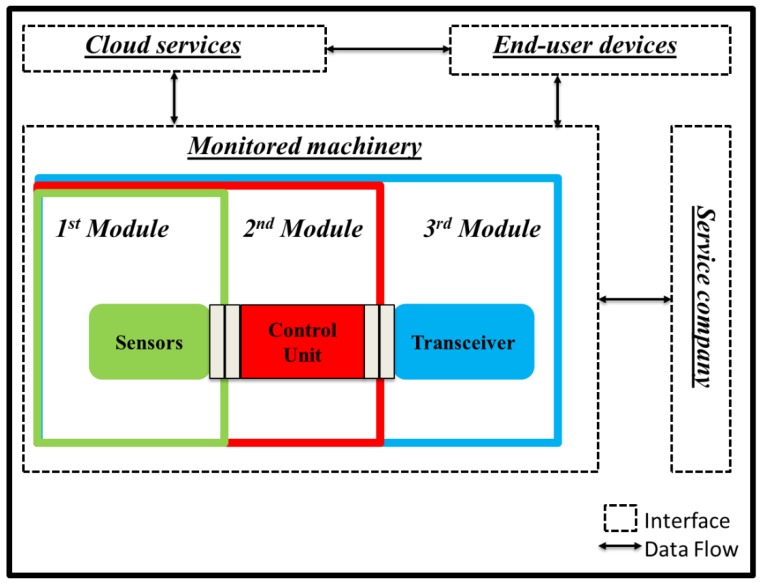
The IoT DAQ for legacy production machinery.

After the steps of Section 4 for threat analysis and application-specific modelling, stage 4 of the proposed approach introduces mitigation mechanisms for priority risks. Retrofitting monitoring solutions on machinery typically involves devices that integrate acquisition, processing, and transmission of data. Such units are compact but may have security shortcomings. In some cases, they employ a single communication protocol for real-time data transmission, which can be restrictive in the sense that if a single communication protocol is compromised, the whole process integrity might be so too. However, increasingly, IoT devices offer multiconnectivity options, which add more flexibility but still the choice of protocol is preset and fixed in most cases. A typical IoT device includes I/O ports for sensing and actuation (1st Module), CPU and memory (2nd Module), communications (3rd Module), and powering options [82]. Each of them in order may be considered to extend the functionality of the previous one, but in integrated IoT devices, their trust boundary encompasses them all together (Figure 10). Such a device can be compromised if any of the three modules is compromised, for example, through cloning. IoT endpoint security can benefit from the IISF principle of component or subsystem isolation and this is adopted here. In contrast to monolithic devices, the proposed design choice is for a modular security approach, by decomposing the overall trust boundary to create a separate trust boundary for each component and implementing security mechanisms in the communication between them.

### The Authentication Protocol

**Figure 11:**
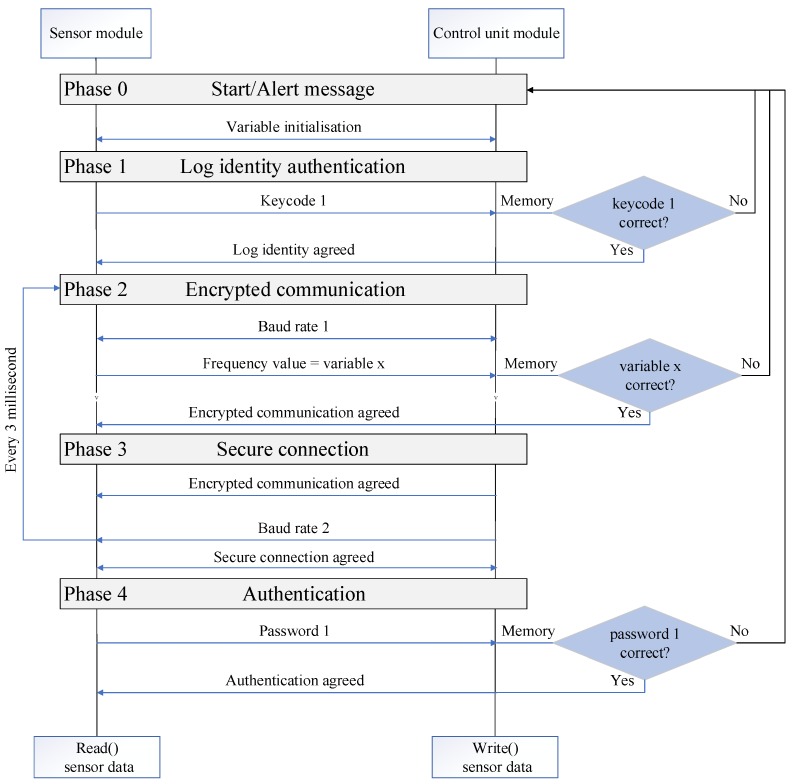
Log identity authentication, encrypted communication, secure connection, and authentication (LCCA) protocol.

**Figure 12:**
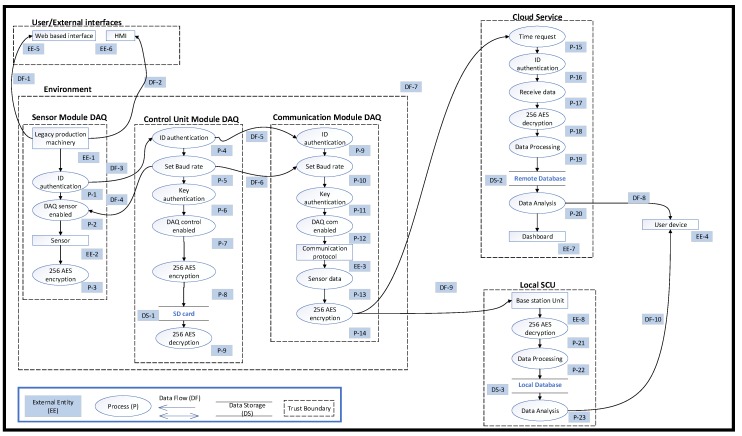
Data flow diagram of the connected legacy production machinery.

The authentication protocol for the modular IoT DAQ is illustrated in Figure 11 and Figure 12. Specifically, the flowchart in Figure 11 shows the process flow, while the DFD in Figure 12 depicts the data flow. The protocol comprises four steps: Log identity authentication, encrypted communication, secure connection, and authentication, and will be referred to as LCCA. All LCCA phases employ AES cryptography. The phases are serially executed and failure to execute one as specified, results in issuing a security alert. The LCCA protocol includes a set of keycodes, passwords, baud rates, and frequency values as part of its mechanism to progress through the four phases and can be applied for the communication between the control module and the two other modules. The LCCA flow is described next for the communication between the sensor and the control unit module (Figure 11).

*Phase 0: Start*

• The system initialises the set of keycodes, passwords, baud rate, and frequency relationships to be used, and then enters a sleep mode (START), waiting for the first connection.

*Phase 1: Log identity authentication*

• This phase handles the log identity between the modules. Specifically, if the control unit module recognises the sensor module log identity, the protocol proceeds, otherwise the process freezes. In our example log identities (and to the same purpose baud rate, frequency values, and passwords) are prestored in a dictionary embedded in each module, but algorithmic approaches to dynamically create them could be employed instead.

*Phase 2: Encrypted communication*

• This step sets an agreed value for the data transfer rate (baud rate) between the control unit and sensor modules. In this way, the two modules engage in a handshake process. The LCCA algorithm sets the initial rate (baud rate 1) and at real-time every fixed time period (in this example, 3 ms) the algorithm changes the control unit rate with a new rate value (baud rate 2), according to (based on frequency x in Figure 11) a formula known in advance between the modules. Upon agreement, data exchange progresses, and all data transfers are encrypted. Any mismatch between the two, which may arise as a result of a security breach, will pause communication and set the system to sleep mode, issuing an alert. Once encrypted communication is established, the process advances to the next stage, otherwise, the connection is closed and returns to phase 1.

*Phase 3: Secured connection*

• This phase covers the connection between the control unit and the sensor module. Once encrypted communication is established, the control module will expect to receive a frequency value from the sensor module to set a new connection rate at predetermined intervals (set here every 3 milliseconds). If the frequency value is recognised by the control unit module, the protocol continues to the next phase, otherwise will pause communication and set the system to sleep mode, issuing an alert. The modules establish connection, and the control module sends the new frequency in a continuous loop employing the baud rate agreed in phase 2.

*Phase 4: Authentication*

• In this phase, the sensor module alphanumeric password is checked by the control unit. An admissible alphanumeric password is a combination of a minimum of eight characters, including lowercase and uppercase, numbers, and symbols. Additional measures prevent using the same password twice; dictionary words, or sequences; usernames or information that might become publicly associated with the user. If the control unit module does not recognise the password, authentication ends unsuccessfully, and the process moves back to step 3.

The DFD of Figure 12 is a detailed version of Figure 6 to illustrate the data flow through the trust boundaries when the IoT device is equipped with the added security provisions. Instead of the single trust boundary around the IoT device, there are now three trust boundaries, one for each module, and an overall boundary is highlighted for the whole machine equipped with the IoT device. Next, an implementation instance of the LCCA mitigation mechanism and its testing are presented.

## 6. Pilot Implementation and Testing

This section describes the final stage of our security design approach and presents an implementation instance focusing on mitigation mechanisms for DoS and clone attacks, which are considered typical threats, are relevant to production environments, and were marked with a high impact score in the earlier analysis. The hypothesis of the experiments is that of a DoS or clone attack succeeding. This could be achieved through a number of intermediate goals, as shown in the relevant attack tree in Section 5. Once successful, the attacks aim to deprive the IoT device of vital resources and to compromise monitoring data. The mitigation mechanisms follow the principle of modularity and the LCCA protocol described in the previous section. The objective of the testing is to assess the ability of the implemented approach to avert these two types of attacks. An industrial DMG NTX 1000 CNC Mill Turn Centre (twin-spindle turning centre with five-axes milling capability) was employed for the experiments. The remainder of this section describes the physical instantiation of the IoT DAQ unit and the mitigation mechanisms testing DoS and cloning attacks.

### 6.1. DoS Attack

**Figure 13:**
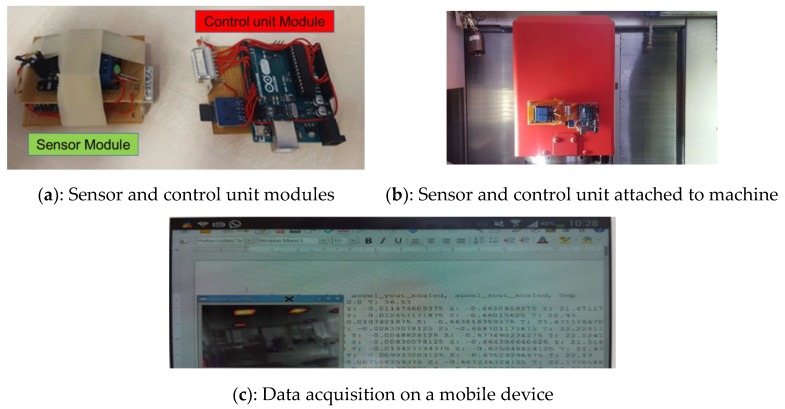
Prototype of modular IoT DAQ.

This section describes the implementation of the mitigation mechanism for the DoS attack. The test was run during the warm-up phase of the machine tool operation. The functional objective was to introduce the IoT DAQ for real-time monitoring of signals, such as acceleration and temperature from the machine spindle, then send the encrypted sensor data to a server (ownCloud) integrated into a raspberry pi 2 model band gain authentication to access and visualise data. The IoT modules are emulated through Arduino Uno units. The attack goal was to generate a DoS situation to jam the IoT device, affecting its battery life and communications, or gain access to monitored machine parameters, such as the spindle temperature and acceleration. The attack tree in Figure 9, shows attack paths that can lead to achieving the target. The modular IoT DAQ is shown in Figure 13. The control unit module is equipped with a 32 GB SD card to store data and its CPU runs the authentication protocol. The sensor module comprises a bottom layer that includes the sensors, the CPU and memory of the control module and the battery to supply the entire IoT unit during data acquisition and protocol execution; and a top layer that includes a relay board to manage data acquisition and apply the mitigation mechanism.

The control unit module is equipped with code to calculate CPU and RAM usage. If the control unit does not identify correct credentials, i.e., valid keycode between the modules, the data acquisition and transmission processes are interrupted, sending an alert message to the user device. A snapshot of the user device screen during monitoring real-time data is shown in Figure 13c, where current data are shared with end-user devices and are visualised. The web-server, cloud, or end-user device are attack points, exposing the monitoring device to a DoS attack aiming to take down its operational capacity.

**Figure 14:**
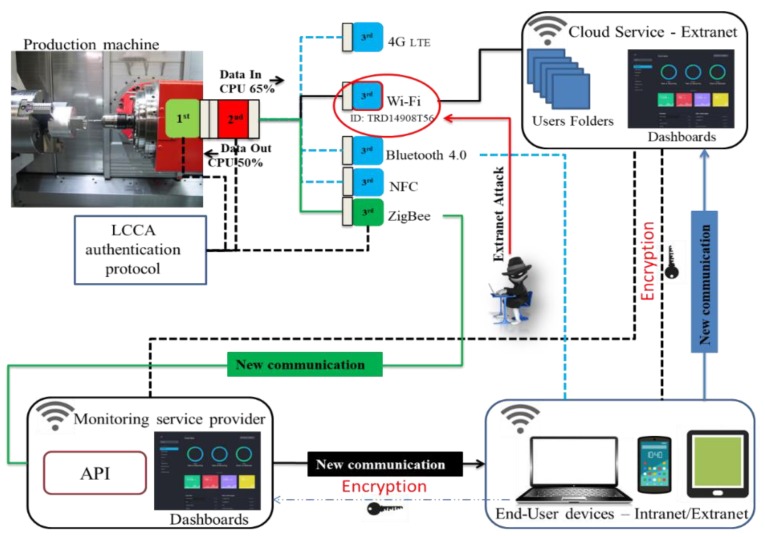
An external DoS attack.

A DoS attack emulation scenario was set up (Figure 14) and includes:

1. The machine tool equipped with the sensor module on the spindle;
2. A hub for a monitoring service provider equipped with an API to make available, through the local network, the machine tool state and performance;
3. End-user devices used to monitor the machine tool anywhere and anytime;
4. The cloud service for processing, analysing, and planning maintenance interventions.

**Figure 15:**
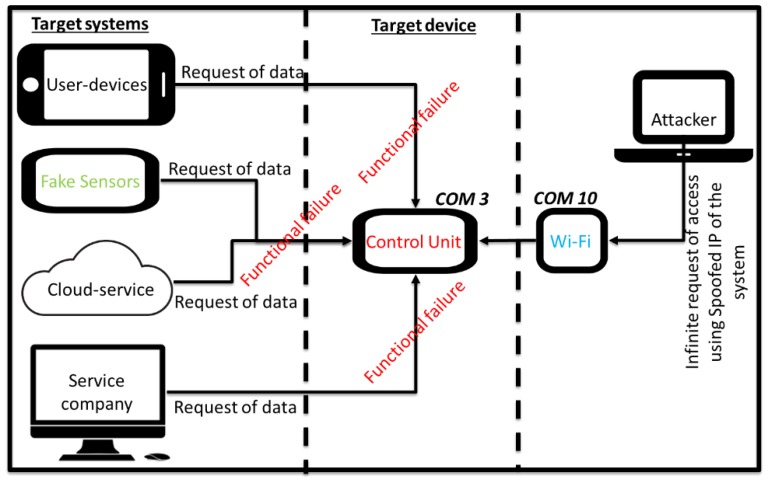
A DoS attack process.

All communications apply AES 256 encryption. The test aimed to simulate a denial of service (DoS) [73] via the network. The attacker gained network access through any of the earlier mentioned methods and is ready to generate connection requests to the communication module using its source address rather than the attack target. In this way, the communication module will respond affirmatively to the connection request not by the attacker but to the target of the attack. The result is a vicious circle that will quickly exhaust the targeted resources and flood the network with traffic (Figure 15). The attack generates an infinite request for access after spoofing the IP of the system through a fake source address and bypassing the firewall. At the same time, the targeted systems attempt to access the data when the sensor module seeks to exchange condition monitoring data with the control module. The large number of responses from the control module causes bandwidth exhaustion and hence a crash. An Arduino Uno was used for generating a connection request to the communication module, so as to affect the targeted device. The control unit module is connected to the sensor module via COM3 port and the communication module through the COM10 port. The attacker, after gaining the network authentication, could take down the capacity of the control module generating an autonomous function, able to generate infinite access requests, delay services, and reduce the battery life.

**Figure 16:**
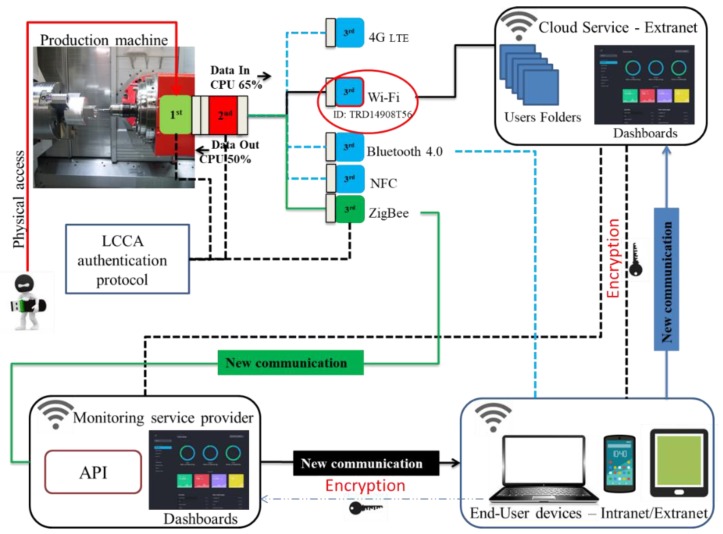
A DoS attack initiated via infected USB dongle.

As a result of this attack, the control unit will output the estimated ratio of CPU usage as shown in Figure 16 for the control unit module:

$$CPU\;utilisation\;\left(\%\right)=\frac{CPU\;Idlecountintime\;frame\;\times \;100\;}{Maximum\;number\;of\;CPU\;Idlecountintime\;frame}$$

The CPU utilisation procedure includes two phases:

- Phase 1: The real-time operating clock (RTOC) is used to estimate CPU/core utilization. The scheduler system tick is used for this purpose, as it is based on timer interrupt, which is considered as a relatively accurately measure of elapsed time.
- Phase 2: Counting maximum idle count; an estimation is obtained through observing idle counts during a measurement period. If no task is performed (besides the timer interrupt) this represents the maximum number of idle counts and corresponds to 0% utilisation. Estimation accuracy errors tend to become insignificant when the CPU utilization measurement period is sufficiently large. After calculation of maximum idle counts, no code or task can be added to the idle task.

The CPU utilisation is contrasted against the expected average value for this device, which in this case was known to be 71% without any attacks. The initialisation stage when starting the CPU generates a level of 22% usage and this is due to a delay of the function printer at the screen. Reaching 100% is a strong indication that the CPU is under attack. In our case, the DoS attack materialises by running our application via a host computer on the intranet. The control unit and transmitter module exchange information using the LCCA protocol (Figure 11) to detect significant deviations from the expected standard operation. If the DoS attack occurs on the current available channel for exchanging data (for example, on the Wi-Fi module circled red in Figure 14), the control module recognises the attack and shuts off the current communication path. The scope of this test was to perform an end to end functional testing without fully emulating any kind of DoS attack or their formal mitigation mechanisms. The aim was to illustrate how the isolation principle is applied through the LCCA protocol to reduce relevant security risks. The simple detection technique can nonetheless be replaced by a more sophisticated mechanism, while following a similar isolation principle in the communication between modules.

### 6.2. Cloning Attack

**Figure 17:**
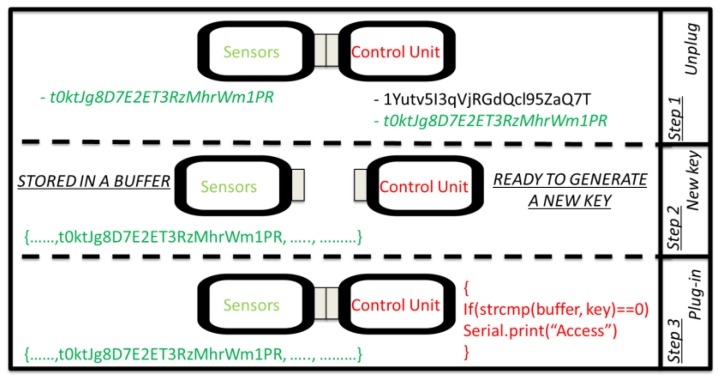
Control unit and sensor; communication module authentication process.

**Figure 18:**
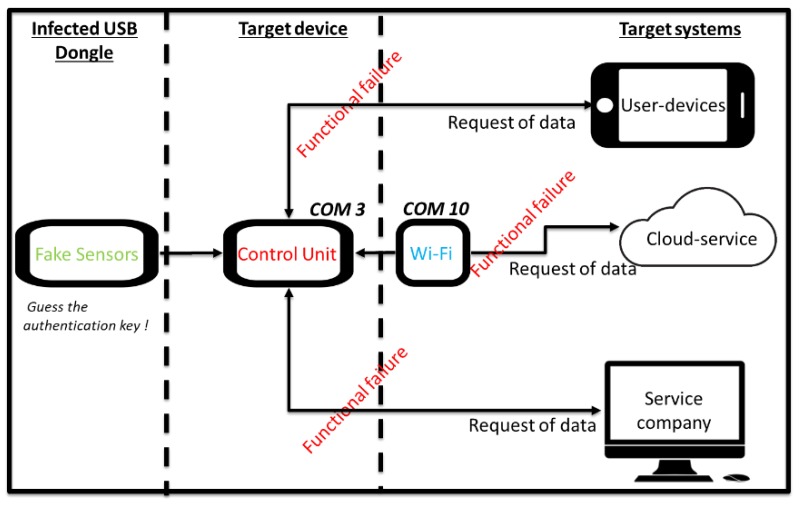
Authentication method vs. the distributed denial of service (DDoS) attack with infected USB dongle.

In the second test, the attacker gains access to the communication module by the social engineering method or via bypassing the firewall. The mitigation mechanism is to use the control unit module to read the authentication key and detect abnormal states. When some of the hardware or software parameters (e.g., voltage, current, system memory, CPU usage, connection buses, or IDs) change in unexpected ways, the control unit module closes the current connection with the malicious hardware or software components and initiates an alternative way for exchanging data with the target. In the scenario of Figure 16, the attacker gains physical access to the sensor module and is able to clone it using fake modules equipped with reprogrammed the firmware. Figure 17 shows the authentication process between two modules of the modular IoT DAQ. For each connection between the modules, the control unit module generates a new unique authentication key (Step 1). The key is stored within the sensor module in a buffer of characters under a private class that does not allow modifications by other users (Step 2). The last phase (Step 3) checks the sensor unique key and compares it to the one in the control unit module buffer. If the sensor unique key matches the key inside the control buffer unit, the sensor module gets access to phase 2 of the authentication protocol (Figure 11). Upon guessing the authentication key, the attacker gains access to the target device and initiates the DoS attack. Figure 18 shows the DoS attack when an infected USB dongle is employed for upgrading an infected kernel inside of the machine [83]. Such an attack may employ multiple attacking nodes, which together form a botnet. A botnet is a network controlled by a master bot and is made up of devices infected by specialised malware, known as bots or zombies [84]. In a cloning attack of a wireless sensor network architecture, once a sensor node is compromised, the adversaries can easily capture other sensor nodes and deploy several clones that have legitimate access to the network (legitimate IDs, passwords, and other security credentials) [85]. The cloning attack affects the mobile communication protocol as well. Subscriber identity module (SIM) cloning by physical access is a simple process and the attacker must have a software program, a SIM reader, and a SIM chip writer [86]. Such examples highlight the risk of cloning attacks, which can be addressed by cryptography or physically unclonable functions (PUFs) [87]. In the IoT DAQ the control unit module is the master that controls all operations and requires protection. The cloning attack involves tampering with the sensor and communication modules, aiming to compromise the architecture integrity and modify the behaviour of the modules.

**Figure 19:**
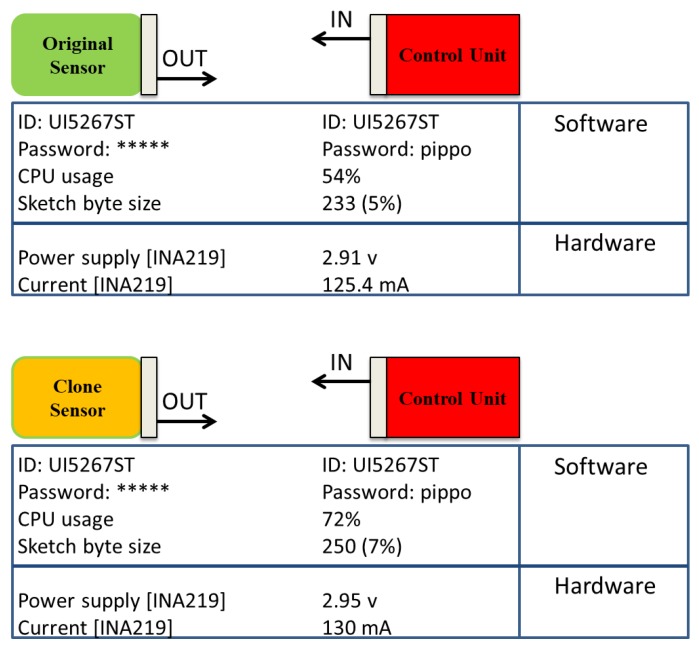
Reading byte between two modules.

Figure 18 illustrates also the mitigation approach effect for the cloning attack in case the attacker guesses the key. The control module analyses data from the sensor and communication modules to detect deviation between the original and the clone sensor behaviour. When the clone sensor is identified, the control module disables all communication with the clone sensor. Figure 19 shows two different cases of sensor communication. In the first case, the control module is connected with the original sensor (green module); reading parameters, such as ID, password, CPU usage, static RAM (SRAM) byte sketch size; and hardware parameters through the INA219 sensor (power supply and current). At the second case (bottom), the clone sensor (amber module) shows the same hardware and software of the original sensor but the malicious code for compromising the monitoring system is also included. To detect signs of a cloning attack, the control module monitors changes in CPU usage, power supply and current, comparing them against typical values. In addition, the control unit reads the sketch byte size to understand the credibility of the sensor module. The sketch byte size is stored into the microcontroller SRAM and show the unique value of the sketch. If the adversary seeks to modify the code to add the malicious part and leave the rest of the sensor module the same as the original, the control module can recognise it as a clone module and will not share any information with it because of the deviation of sketch byte size and level of usage of SRAM. Physical parameters can help to single out unexpected changes to hardware parameters. The authentication ID control mechanism brings the probability of successful cloning threat events to a lower level, reducing the impact score rating.

## 7. Discussion and Related Future Work

The large amount of interconnected things for advance manufacturing brings new cyber risks. Production environments are strongly characterised by jointly involving OT and IT and the potential impact of any security breaches on the integrity of industrial systems can be very tangible and highly critical. Cyber security must therefore be a vital part of the design, operations, and strategy processes, and should be considered from the very beginning of any new connected Industry 4.0 driven initiative.

The reported work introduced a systematic design thinking approach to attack the new risks arising with the Industry 4.0 connectivity. The new approach draws parallels with previous and ongoing activities (e.g., PASTA, IISF, ENISA) but is positioned towards the concrete context of retrofitting legacy production machinery with IoT-enabled monitoring capabilities all the way from the study of requirements, and through threat and application modelling, all the way to threat mitigation design, implementation, and testing with prime focus on IoT endpoint security. As an exemplar of dealing with this challenge, this paper introduced a new security hardware IoT device for remote monitoring application in a production environment, which is managed through a flexible but strong lightweight authentication protocol and mechanisms for isolation between the key subsystems of an IoT endpoint device. This was tested through a real-world case study where security flaws were deliberately introduced, a qualitative risk assessment was applied, and relevant risks were mitigated.

Overall, the main paper contribution is in the overall design thinking approach, while several additional contributions which were included, such as the new authentication protocol implementing in effect the isolation principle at the IoT endpoint subsystem level. However, the principle of subsystem isolation goes well beyond physical or subsystem interfaces isolation. IoT deployments now make extensive use of containerisation technologies and IoT devices can themselves be put under this context through container engines and container APIs [88], which can link endpoint devices to an extended system of IoT-enabled application services [89]. Significant ongoing research currently targets the extension of IoT resources orchestration to jointly include both edge nodes and the cloud [90]. Based on the above, the research presented in this paper opens up several threads for further work:

- Comprehensive mitigation mechanisms for the range of identified threats. While the reported work presented an implementation example of the design thinking approach, which included specific instances of mitigation mechanisms relevant to preventing DoS and clone attack threats, any alternative and more comprehensive mechanism can be employed instead but would still need to be included within the context of an overall design approach for IoT security.
- Eventually, any introduced mitigation mechanism needs to be scrutinised for effective protection against attacks. The reported work intention was to present the multistage design thinking approach, with mitigation being a concrete step within this. Any final deployment of adopted solutions needs to be preceded by extensive and systematic testing against attacks. Such a testing will need to consider simulation or indeed emulation of attacks, as well as mechanisms for their systematic generation [90].
- The reported work includes risk assessment and mitigation as part of the five-stage systematic approach. However, risk quantification was only indicative and of qualitative nature. Further work is needed in the direction of systematic risk quantification, including approaches for data- and evidence-driven risk quantification [91]. While this is highly important for IoT endpoint devices, overall IoT network security is only as good as its weakest link and a weak node may have scalable negative impacts to the whole IoT network. Further work needs to put into such a context any risk-based approach to security and duly take into account complexity considerations.
- The isolation principle in IoT is effectively applied through virtualisation and containerisation technologies, as expressed, for example, by the IISF. While such technologies were more applicable to cloud services, they are increasingly expanded and implemented at the edge node level. IoT endpoint device security can strongly benefit via joint physical and virtual isolation, and future research need to align relevant research with such IoT architecture patterns [89,90].
- Organisations seeking to adopt security-by-design approaches would benefit from methodologies and tools that assist in appropriate prioritisation of any upgrades related to security. It is futile to implement the most sophisticated approach for part one aspect of security, when others are left too weak. Maturity assessment methods and tools are helpful to this end. Future work would need to look how to best place a design thinking approach, such as the one presented in this paper, within the context of overall organisational security maturity management [92].

## 8. Conclusions

This paper introduced a novel endpoint security design approach to address security issues when upgrading production machinery with IoT connectivity to deliver real-time condition monitoring for legacy production machinery. The approach considers best practice and guidelines to formulate a new domain-specific approach, contributing to bridging the gap between introducing IoT connectivity at the shop floor and shielding system and operational integrity. The main concepts of the new approach are the application-aware viewpoint, as opposed to generic security measures, the adoption of the principle of subsystem isolation, and the development of a new multistage but lightweight authentication protocols, which are all contributing to increasing the required complexity of any attack approach to achieve compromising the IoT device and associated monitoring and production processes. The concrete implementation of this approach was demonstrated through two industrial legacy machinery attack scenarios based on different attack entry points, for DoS and cloning attacks. The approach enables the mapping and prioritisation of threats and risks in a domain-specific application-oriented way, which, in turn, allows the identification of priorities for intervening with mitigation approach and lowers integrity risks.

While the new approach and its implementation focuses on the key design aspects, rather than on any single sophisticated detection mechanism, it is worth noticing that the employed mechanisms can be upgraded to introduce stronger detection, and therefore, response capabilities. Future research needs to target such capabilities but will need to develop a systematic approach for testing. The risk-based part of the methodology needs to evolve further from qualitative to quantitative, and be linked to the results of the testing phase to improve security performance. Production environments are considerably different from others due to the dominant presence of OT, which may imply significant operational impacts. It is for this reason, that dedicated testbeds and domain-specific security metrics need to be developed and employed in a systematic testing and evaluation process, while for the detection and response mechanism, other sophisticated algorithmic and other approaches could be used as part of the overall methodology for IoT device endpoint security protection. Overall, this paper included a discussion with leads to further research (Section 7), pointing out the need for further research in the direction of (a) comprehensive mitigation mechanisms; (b) systematic test generation and validation of solutions; (c) automated and data-driven risk assessment; (d) impact of endpoint vulnerabilities on overall IoT network security; (e) virtual isolation, IoT edge node containerisation and virtual–physical nodes orchestration; (f) systematic maturity assessment and management for IoT security.

## Figures and Tables

**Table 1:** An example of threat analysis. HIT: Human interaction; ATT: Advanced technical; PT: Physical threats.

Activity Description	Impact Examples	Countermeasure Mechanisms	Threat Types		
			HIT	ATT	PT
1. Negligence Errors and vulnerabilities linked to the launch of a new network within a production environment.	Network delays or errors lead to poor control or loss of control over certain production processes	Operations procedures to be followed by personnel installing or using the network	x		
2. Social Engineering Uses the human behaviour to gain security access without the victim realising the manipulation.	Could cause system integrity loss (e.g., data loss or tampering, system process malfunctions, poor product quality, health and safety issues).	Training about the social engineering threat, company policy, and procedures.	x		
3. Denial of service (DoS) Channel is flooded with data, exhausting bandwidth.	Breakdown of network control, causing loss of production monitoring and control capabilities	Network traffic analysis and detection systems		x	

**Table 2:** Impact rating.

**High (H)**	The Threat Is Unacceptable and Immediate Measures Are Needed to Reduce It to Preserve Data or System Integrity.
**Medium (M)**	The threat may be acceptable over the short term but countermeasures to reduce the risk should be implemented.
**Low (L)**	The risks are acceptable. Measures to reduce risk can be taken in conjunction with other actions, for example, during upgrades.

**Table 3:** Risk likelihood (chance rating).

**High (H)**	A Highly Motivated and Sufficiently Capable Threat-Source; Protection Countermeasures Are Ineffective.
**Moderate (M)**	The source of the threat is motivated and capable, but some countermeasures in the short term could hinder the success of attacks.
**Low (L)**	Limited motivation and capability of threat-source; the countermeasures are sufficient to prevent the hazard.

**Table 4:** Score rating (SR) = Impact rating × Chance rating.

Impact → →				
**Chance →→**		**Low (L)**	**Moderate (M)**	**High (H)**
	**High (H)**	L × H = M	M × H = H	H × H = H
	**Moderate (M)**	L × M = L	M × M = M	H × M = H
	**Low (L)**	L × L = L	M × L = L	H × L = M

**Table 5:** Threats classification for IoT-enabled production environments.

Activity	Threat Types			External Entity (EE)	Data Flow (DF)	Data Store (DS)	Process (P)	Impact Rating	Chance Rating	Score Rating
	HIT	ATT	PT							
Negligence	X						X	M	M	M
Social Engineering	X			X			X	H	L	M
Tampering			X		X	X	X	H	L	M
Physical Intrusions	X		X		X	X	X	H	L	M
User Misuse	X			X			X	H	L	M
Unauthorised remote accesses	X						X	H	L	M
External hardware	X						X	H	L	M
Physical destruction	X		X				X	H	L	M
Command injection		X			X		X	M	L	L
Denial of Service (DoS)		X			X	X	X	H	M	**H**
Signal replaying		X			X	X	X	M	L	L
Cloning		X			X	X	X	H	M	**H**
Remote switch off		X			X		X	H	L	M
Signal blocking or jamming		X			X		X	H	L	M
Reverse engineering		X	X		X	X	X	H	L	M
Side-channel		X	X		X	X	X	H	L	M
Wireless zapping		X			X		X	M	L	L
Software compromise	X	X			X	X	X	H	L	M
Electromagnetic interference			X				X	M	L	L
Cable cuts			X	X			X	H	L	M
Power fluctuation			X				X	M	M	M
Voltage spikes			X				X	H	L	M
Installation errors			X				X	M	L	L
Takeover of an authorised session			X	X	X	X	X	H	L	M

**Table 6:** Attack goals and impacts. P: Primary; S: Secondary; I: Information; U: Interface.

Attack Goal	Impact Description	Function Code
Network access	Inability to communicate with the DAQ	I, U
	Inability to communicate with the Cloud	U
	Inability to communicate with the SCU	C, U
	Inability to communicate with User Devices	I, U
	Inability to upgrade firmware	I
System communication access	Inability to use the HMI	I, U, P
	Inability to use the DAQ modules	I, C
	Inability to use the legacy production machinery	P, S
DAQ access	Inability to collect correct sensor data	C, I
	Inability to protect sensor data	C, I
	Inability to send data correctly	C, I, U

## Data Access

Data underlying the testing can be accessed at https://doi.org/10.17862/cranfield.rd.8143340

## Abbreviations

AES	Advanced Encryption Standard
CBM	Condition-Based Maintenance
CM	Condition Monitoring
CNC	Computer Numerical Control
CPS	Cyber-Physical Systems
CPPS	Cyber Physical Production Systems
CPU	Central Processing Unit
DAQ	Data Acquisition
DFD	Data Flow Diagram
DoS	Denial of Service
DTLS	Datagram Transport Layer Security
HMI	Human Machine Interface
ICS	Industrial Control Systems
IT/OT	Information Technology/Operational Technology
IoT	Internet of Things
MAC	Medium Access Control
MITM	Man-in-the-Middle
LCCA	Log identity, Communication, Connection, Authentication
OT	Operational Technology
P2P	Peer-to-Peer
PAC	Programmable Automation Controller
PLC	Programmable Logic Controller
PLM	Product Lifecycle Management
RMS	Remote Monitoring System
SCADA	Supervisory Control and Data Acquisition
SCU	System Control Unit
SQL	Structured Query Language
WSN	Wireless Sensor Network
